# The prediagnostic period of Behçet’s disease: a nationwide population-based study

**DOI:** 10.3389/fimmu.2026.1970448

**Published:** 2026-10-14

**Authors:** Fadi Hassan, Jawad Abu-Elhija, Areej Bayatra, Yolanda Friedrich, Mohammad E. Naffaa, Ariel Israel

**Affiliations:** 1 Rheumatology Unit, Galilee Medical Center, Nahariya, Israel; 2 The Azrieli Faculty of Medicine, Bar-Ilan University, Safed, Israel; 3 Leumit Research Institute, Leumit Health Services, Tel Aviv, Israel; 4 Department of Epidemiology and Preventive Medicine, School of Public Health, Grey Faculty of Medical and Health Sciences, Tel Aviv University, Tel Aviv, Israel

**Keywords:** Behçet disease, early diagnosis, inflammation, prediagnostic footprint, prediagnostic period

## Abstract

**Introduction:**

The diagnosis of Behçet’s disease (BD) is often delayed because its characteristic manifestations may emerge sequentially over time. Whether BD is preceded by a clinically active prediagnostic phase remains unclear. We aimed to characterize the clinical and laboratory features during the five-year period preceding the formal diagnosis of BD.

**Methods:**

We conducted a nationwide retrospective matched case–control study using the electronic health records of Leumit Health Services. Patients with BD fulfilling the International Criteria for Behçet’s Disease or International Study Group classification criteria were identified by manual chart review and matched 1:5 with controls by sex, age, socioeconomic category and year of first observation. Clinical diagnoses, diagnostic investigations and laboratory measurements and medication dispensing recorded during the five years preceding diagnosis were compared between groups. Early and late laboratory measurements within the prediagnostic period were analyzed to assess temporal trends. Manifestations documented over the entire available record were analyzed separately.

**Results:**

The study included 218 patients who subsequently developed BD and 1,090 matched controls. During the five years preceding diagnosis, future patients with BD more frequently exhibited oral aphthosis (36.2% vs 3.0%; OR 18.2), panuveitis (4.6% vs 0.1%; OR 52.4), inflammatory polyarthritis (7.3% vs 0.6%; OR 14.3), and venous thromboembolism (4.6% vs 0.2%; OR 26.2) (all P<0.001). Over the entire available record, oral aphthosis was documented in 61.0% of patients with BD and 11.8% of controls. Autoimmune serology was frequently requested but remained largely negative. Laboratory analyses demonstrated a biphasic trajectory. Absolute monocyte count and the neutrophil-to-lymphocyte ratio were lower than in controls at the early assessment (0.40 vs 0.44 ×10^9^/L, P = 0.009, and 1.45 vs 1.49, P = 0.023) but significantly higher near diagnosis (0.64 vs 0.58 ×10^9^/L, P = 0.025, and 2.44 vs 2.09, P = 0.003). This transition was accompanied by rising CRP, ESR, platelet count and globulin levels, persistently lower mean corpuscular volume and consistently higher IgA at both assessments (247 vs 189 mg/dL, P = 0.009, and 256 vs 189 mg/dL, P = 0.012), whereas IgG and IgM remained comparable between groups.

**Conclusions:**

BD seems to be preceded by a clinically and biologically active prediagnostic phase characterized by a distinct inflammatory multisystem footprint.

## Introduction

Behçet’s disease (BD) is a chronic, relapsing, multisystem disease characterized by recurrent mucocutaneous ulceration together with variable ocular, articular, vascular, gastrointestinal and neurological involvement (1, 2). Although mucocutaneous manifestations are the most common features, major organ involvement may result in substantial morbidity and even mortality, such as irreversible visual loss from posterior uveitis and retinal vasculitis, venous thrombosis and arterial aneurysms (1–4). BD is thought to result from interactions between genetic susceptibility, most notably HLA-B51, environmental factors, and dysregulated innate and adaptive immune responses (5, 6). Despite increasing understanding of the pathogenesis, no biomarker has sufficient diagnostic accuracy for routine clinical diagnosis (7–9).

Consequently, the diagnosis of BD remains primarily clinical, as no pathognomonic laboratory, imaging or histological test is available. The International Study Group (ISG) and International Criteria for Behçet’s Disease (ICBD) are widely used classification criteria that may support disease recognition (9, 10). The ISG criteria require recurrent oral ulceration together with at least two additional characteristic manifestations, whereas the ICBD use a weighted scoring system incorporating mucocutaneous, ocular, neurological, vascular, and pathergy findings (10, 11).

Importantly, the characteristic manifestations of BD often emerge sequentially rather than simultaneously, and patients may therefore remain with incomplete or nonspecific phenotype for prolonged periods before sufficient clinical features accumulate to justify disease recognition (9). This may delay appropriate treatment and allow ongoing inflammation that results in irreversible organ damage. Importantly, the prediagnostic period may be characterized by repeated healthcare encounters, investigations, specialist referrals, treatment for individual manifestations, and alternative diagnostic labels (1, 9, 12, 13). Such a trajectory is increasingly recognized in other immune-mediated diseases, including rheumatoid arthritis, systemic lupus erythematosus, inflammatory bowel disease, and systemic sclerosis, where clinical, laboratory, and healthcare-utilization changes may precede diagnosis by months or even years (14–20). Whether BD follows the prediagnostic pattern remains unclear (21). Previous studies, primarily from Turkey, Iran, and Japan, have largely examined the chronology of overt manifestations in an already-diagnosed cohorts, demonstrating that oral aphthosis and other mucocutaneous manifestations may precede diagnosis by several years, whereas gastrointestinal, neurological, and vascular involvement generally occurs later (22–26). However, the broader prediagnostic trajectory of BD, including evolving clinical manifestations, laboratory abnormalities, healthcare utilization, alternative diagnostic labels and treatment patterns before diagnosis, remains poorly characterized.

BD is well recognized in Israel, although its prevalence varies substantially across ethnic groups. A population-based study reported an overall prevalence of 15.2 per 100,000, ranging from 8.6 per 100,000 among Jewish individuals and 26.2 per 100,000 among Arabs to 146.4 per 100,000 among Druze (27). This ethnically diverse population, coupled with comprehensive longitudinal electronic health records, provides a unique opportunity for investigating the prediagnostic evolution of BD. Large-scale electronic healthcare databases enable the assessment of routinely collected longitudinal clinical data, minimizing recall bias and extending investigation beyond traditional referral-center cohorts (28–30). By integrating clinical diagnoses, diagnostic procedures, laboratory measurements, healthcare utilization, and matched population controls, such databases allow multidimensional characterization of the prediagnostic period that is difficult to achieve in conventional single-center cohorts.

Therefore, we aimed to characterize the five-year prediagnostic period preceding the diagnosis of BD using a nationwide population-based electronic healthcare database. Specifically, we investigated the evolution of disease-related clinical manifestations, diagnostic investigations and longitudinal laboratory trajectories.

## Methods

### Study design

We conducted a retrospective matched case–control study using the electronic medical records of Leumit Health Services (LHS), one of the four nationwide health maintenance organizations in Israel. LHS provides comprehensive healthcare to a socioeconomically and geographically diverse population and maintains a centralized longitudinal database containing demographic information, physician diagnoses, laboratory results, medication prescriptions, healthcare utilization records and diagnostic procedures. The validity of the LHS database for epidemiological and clinical research has been demonstrated in previous studies (31, 32).

### Study population

Patients with BD were identified using the International Classification of Diseases, Ninth Revision (ICD-9) diagnostic code for Behçet’s disease (“136.1”), the coding system used throughout Israel (**Figure 1**). To confirm the diagnosis of BD, all potential cases underwent independent manual chart review by two board-certified rheumatologists with expertise in diagnosing and treating BD (F.H. and M.E.N.). The diagnosis of BD was confirmed only if patients fulfilled either the ICBD or ISG classification criteria. Patients who did not satisfy either set of classification criteria after manual review were excluded from the study. Patients with a qualifying BD diagnosis between January 1, 2003 and December 31, 2025 were eligible for inclusion. For each confirmed case, the index date was defined as the earliest qualifying BD diagnosis recorded in the LHS community or hospital data, and clinical information during the preceding five years was analyzed.

**Figure 1:**
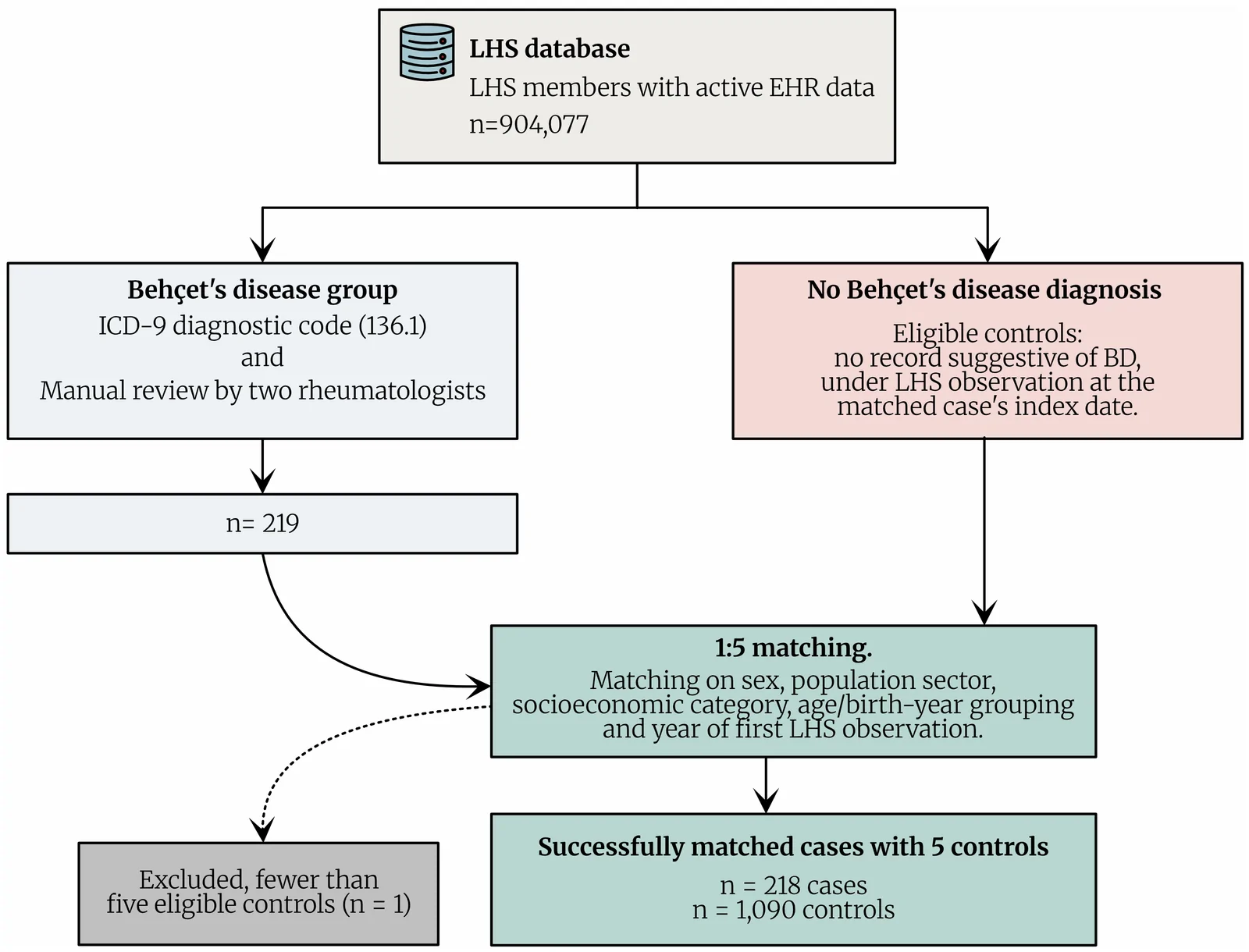
Study flow diagram illustrating identification and selection of the study population. Flowchart summarizing the identification and selection of patients with Behçet’s disease (BD) and matched controls from the Leumit Health Services electronic health records database. Among 904,077 individuals with active electronic health record data, patients with an ICD-9 diagnosis of Behçet’s disease (code 136.1) underwent independent manual chart review by two rheumatologists to confirm fulfillment of the International Criteria for Behçet’s Disease or International Study Group classification criteria. Of 219 eligible patients, 218 were successfully matched to five controls each according to age, sex, enrollment year, socioeconomic status, and ethnicity, resulting in a final study population of 218 patients with Behçet’s disease and 1,090 matched controls. BD, Behçet’s disease; EHR,electronic health record; ICD-9, International Classification of Diseases, Ninth Revision; LHS, Leumit Health Services.

Controls were selected algorithmically from the general LHS membership population, rather than from rheumatology or other specialty clinics, using a 1:5 matched-set design. Individuals with community or hospital records suggestive of BD were excluded from the control pool, whereas controls were not required to be free of other medical conditions. Matching incorporated sex, population sector, socioeconomic category, age/birth-year grouping, and year of first LHS observation, with nearest matching on date of birth. An iterative matching procedure progressively relaxed the coarsening and distance caliper when necessary to identify five eligible controls while preserving the predefined matching dimensions. Controls were additionally required to be under observation in LHS at the index date of their matched case and were assigned the same index date, allowing an identical patient-relative pre-index observation period to be applied to cases and controls. Propensity-score matching was not used. Of the 219 confirmed eligible BD cases, 218 were successfully matched to five controls, resulting in 218 cases and 1,090 controls. For both groups, all available clinical information recorded during the five years preceding the index date was extracted for analysis.

### Study variables

Demographic characteristics included age, sex, ethnicity, socioeconomic status, geographic district, calendar year of the index date, body weight, body mass index (BMI), smoking status, hypertension, and diabetes mellitus. Clinical manifestations recorded during the five-year prediagnostic period were identified using ICD-9 diagnostic codes and categorized according to organ system, including mucocutaneous, ocular, musculoskeletal, vascular, gastrointestinal, and infection-related manifestations. For each clinical manifestation, patients were classified as having the manifestation if at least one corresponding diagnosis was recorded during the five-year prediagnostic interval. These frequencies therefore represent cumulative documented occurrence within the predefined five-year window rather than the first manifestation or lifetime cumulative phenotype of BD. To characterize the cumulative phenotype separately, the same manifestations were additionally assessed over the entire available LHS record of each individual, including the periods before and after the index date. The frequency of autoimmune, infectious, and genetic investigations performed during the prediagnostic period was assessed. Autoimmune investigations included antinuclear antibodies (ANA), rheumatoid factor (RF), anti-cyclic citrullinated peptide antibodies (anti-CCP), antineutrophil cytoplasmic antibodies (ANCA) and Human Leukocyte Antigen B51 (HLA-B51) testing. Infectious investigations included serological testing for herpes simplex virus (HSV), cytomegalovirus (CMV), Epstein-Barr virus (EBV), hepatitis C virus, and additional infectious agents. For each investigation, both the frequency of testing and the proportion of positive results among tested individuals were evaluated. Routine laboratory measurements performed during the five-year prediagnostic period were extracted from the electronic medical records. To evaluate temporal changes before diagnosis, two predefined time points were analyzed for each laboratory parameter: the first available measurement within the five-year prediagnostic window (“early”) and the last available measurement before the index date (“late”). Laboratory variables included inflammatory markers, complete blood count and differential, platelet indices, iron studies, protein fractions, immunoglobulins, and routine biochemical parameters. Medication exposure during the five-year prediagnostic interval was assessed using pharmacy dispensing records. Exposure was defined as at least one dispensing within the interval. For exposed patients, the time between the first dispensing and the index date was recorded.

### Statistical analysis

Continuous variables are presented as means with standard deviations (SD) or medians with interquartile ranges (IQR), as appropriate, whereas categorical variables are presented as frequencies and percentages. Comparisons between patients with BD and matched controls were performed using t-test or the Mann–Whitney U test for continuous variables and the χ² test or Fisher’s exact test for categorical variables, as appropriate. Odds ratios (ORs) with 95% confidence intervals (CIs) were calculated. For laboratory analyses, early and late measurements were compared between patients with BD and controls at each time point. When multiple laboratory measurements were available during the five-year prediagnostic period, the first recorded measurement and the last recorded measurement before the index date were analyzed. All statistical tests were two-sided, and a P value <0.05 was considered statistically significant. Statistical analyses were performed using R (R Foundation for Statistical Computing, Vienna, Austria).

### Ethical approval

The study was approved by the Institutional Review Board of LHS (0030-23-LEU).

## Results

### Study population and baseline characteristics

The study included 218 patients with BD and 1,090 matched controls. The groups were comparable with respect to age, sex, ethnicity, socioeconomic status, geographic district and calendar year of the index date (**Table 1**). Patients who subsequently developed BD have lower mean BMI than controls (24.53 ± 5.12 vs 26.09 ± 5.81 kg/m²; P<0.001), whereas smoking status and the prevalence of hypertension and diabetes mellitus did not differ significantly between groups (**Table 1**).

**Table 1:** Baseline characteristics of the study population.

Characteristic	Behçet (n=218)	Controls (n=1090)	OR/Mean difference	P value
Age	34.16 ± 13.04	34.15 ± 13.04		0.99
Gender, n (%)				
Male	102 (46.8%)	510 (46.8%)	1.00	1.00
Female	116 (53.2%)	580 (53.2%)	1.00	1.00
Socioeconomic status (1-20)	7.734 ± 3.476	7.698 ± 3.436		0.89
Geographic district, n (%)				
Center	59 (27.1%)	257 (23.6%)	1.20	0.30
Jerusalem	30 (13.8%)	169 (15.5%)	0.87	0.61
North	87 (39.9%)	407 (37.3%)	1.11	0.49
South	42 (19.3%)	257 (23.6%)	0.77	0.19
Calendar year of the index date, mean ± SD	2014 ± 6	2014 ± 6		1.00
Ethnicity				
Arab	95 (43.6%)	475 (43.6%)	1.00	1.00
Jewish	97 (44.5%)	481 (44.1%)	1.01	0.94
Orthodox jewish	26 (11.9%)	134 (12.3%)	0.97	1.00
Weight (kg), mean ± SD	68.20 ± 17.05	71.99 ± 19.72		<0.05
BMI (kg/m2), mean ± SD	24.53 ± 5.12	26.09 ± 5.81		<0.001
BMI, n (%)				
<18.5 Underweight	24 (11.0%)	71 (6.77%)	1.70	< 0.05
18.5-24.9 Normal	105 (48.2%)	406 (38.7%)	1.47	< 0.05
25-29.9 Overweight	63 (28.9%)	334 (31.9%)	0.87	0.42
>=30 Obese	26 (11.9%)	237 (22.6%)	0.46	<0.001
Missing	0 (0.00%)	42 (3.85%)	N/A	N/A
Smoking, n (%)				
Non-smoker	161 (74.5%)	745 (75.1%)	0.971	0.86
Past smoker	1 (0.46%)	15 (1.51%)	0.303	0.33
Smoker	54 (25.0%)	232 (23.4%)	1.092	0.60
Missing	2 (0.92%)	98 (8.99%)	0.094	<0.001
Hypertension, n (%)	16 (7.34%)	78 (7.16%)	1.028	0.89
Diabetes mellitus, n (%)	4 (1.83%)	44 (4.04%)	0.444	0.164

OR, odds ratio; SD, standard deviation; BMI, body mass index; N/A, not applicable.

### Clinical manifestations documented during the prediagnostic interval

During the five years preceding diagnosis, patients who subsequently developed BD exhibited a greater burden of disease-related manifestations across multiple organ systems than matched controls (**Table 2** and **Figure 2**). Oral aphthosis was present in more than one-third of future BD patients (36.2% vs 3.0%; P<0.001). Genital ulceration occurred exclusively among future BD patients (1.4% vs 0%; P = 0.005). Folliculitis (6.0% vs 2.0%) and erythema nodosum (2.3% vs 0.2%) were also more frequent (both P ≤ 0.004). Ocular inflammatory diseases were more common in the prediagnostic period. Anterior uveitis (3.7% vs 0.2%), posterior uveitis (2.3% vs 0%), panuveitis (4.6% vs 0.1%), and retinal inflammation (3.7% vs 0.1%) were rare or absent among controls but occurred more frequently among future BD patients (all P<0.001).

**Table 2:** Clinical manifestations documented during the 5-year prediagnostic period.

Diagnosis	ICD-9 grouping/code	Behçet (n=218)	Controls (n=1090)	OR (95% CI)	P value
Mucocutaneous					
Any oral aphthosis	528.2	79 (36.2%)	33 (3.0%)	18.20 (11.69-28.35)	<0.01
Genital ulceration	616.5	3 (1.4%)	0 (0%)	N/A	0.005
Folliculitis	704.80	13 (6.0%)	22 (2.0%)	3.08 (1.53-6.21)	0.004
Erythema nodosum	695.2	5 (2.3%)	2 (0.2%)	12.77 (2.46-66.26)	0.002
Ocular					
Anterior uveitis	364.000	8 (3.7%)	2 (0.2%)	20.72 (4.37-98.28)	<0.001
Posterior uveitis	363.202	5 (2.3%)	0 (0%)	N/A	<0.001
Panuveitis	360.12	10 (4.6%)	1 (0.1%)	52.36 (6.67-411.20)	<0.001
Retinal inflammation	363	8 (3.7%)	1 (0.1%)	41.49 (5.16-333.45)	<0.001
Musculoskeletal					
Arthralgia involving multiple sites	719.49	24 (11.0%)	21 (1.9%)	6.30 (3.44-11.54)	<0.001
Unspecified polyarthropathy/polyarthritis	716.5	16 (7.3%)	6 (0.6%)	14.31 (5.53-37.01)	<0.001
Sacroiliitis	720.2	8 (3.7%)	1 (0.1%)	41.49 (5.16-333.45)	<0.001
Vascular					
Phlebitis and thrombophlebitis	451	10 (4.6%)	6 (0.6%)	8.69 (3.12-24.16)	<0.001
DVT/other venous thromboembolism	453	10 (4.6%)	2 (0.2%)	26.15 (5.69-120.23)	<0.001
Vasculitis	446	5 (2.3%)	0 (0.0%)	56.19 (3.10-1019.92)	<0.001
Gastrointestinal					
Abdominal pain, any site	789.0	100 (45.9%)	356 (32.7%)	1.75 (1.30-2.35)	<0.001
Diarrhea	787.91	31 (14.2%)	89 (8.2%)	1.86 (1.20-2.89)	0.007
Gastroesophageal reflux disease	530.810	20 (9.2%)	17 (1.6%)	6.38 (3.28-12.39)	<0.001
Gastrointestinal hemorrhage	578	7 (3.2%)	7 (0.6%)	5.13 (1.78-14.79)	0.004
Iron-deficiency anemia, any code	280	37 (17.0%)	85 (7.8%)	2.42 (1.59-3.67)	<0.001
Infection-labelled diagnoses					
Herpes simplex, any code	054	24 (11.0%)	35 (3.2%)	3.73 (2.17-6.41)	<0.001
Herpes zoster, any code	053	3 (1.4%)	10 (0.9%)	1.51 (0.41-5.52)	0.46
Acute tonsillitis, any code	463	85 (39.0%)	281 (25.8%)	1.84 (1.36-2.49)	<0.001
Acute sinusitis, any code	461	34 (15.6%)	101 (9.3%)	1.81 (1.19-2.75)	0.007
Cellulitis and abscess, other sites	682	30 (13.8%)	56 (5.1%)	2.95 (1.84-4.71)	<0.001
Gastroenteritis	558.94	41 (18.8%)	139 (12.8%)	1.58 (1.08-2.33)	0.023

ICD-9, International Classification of Diseases, 9th revision; OR, odds ratio; CI, confidence interval; N/A, not applicable; DVT, Deep venous thrombosis.

**Figure 2:**
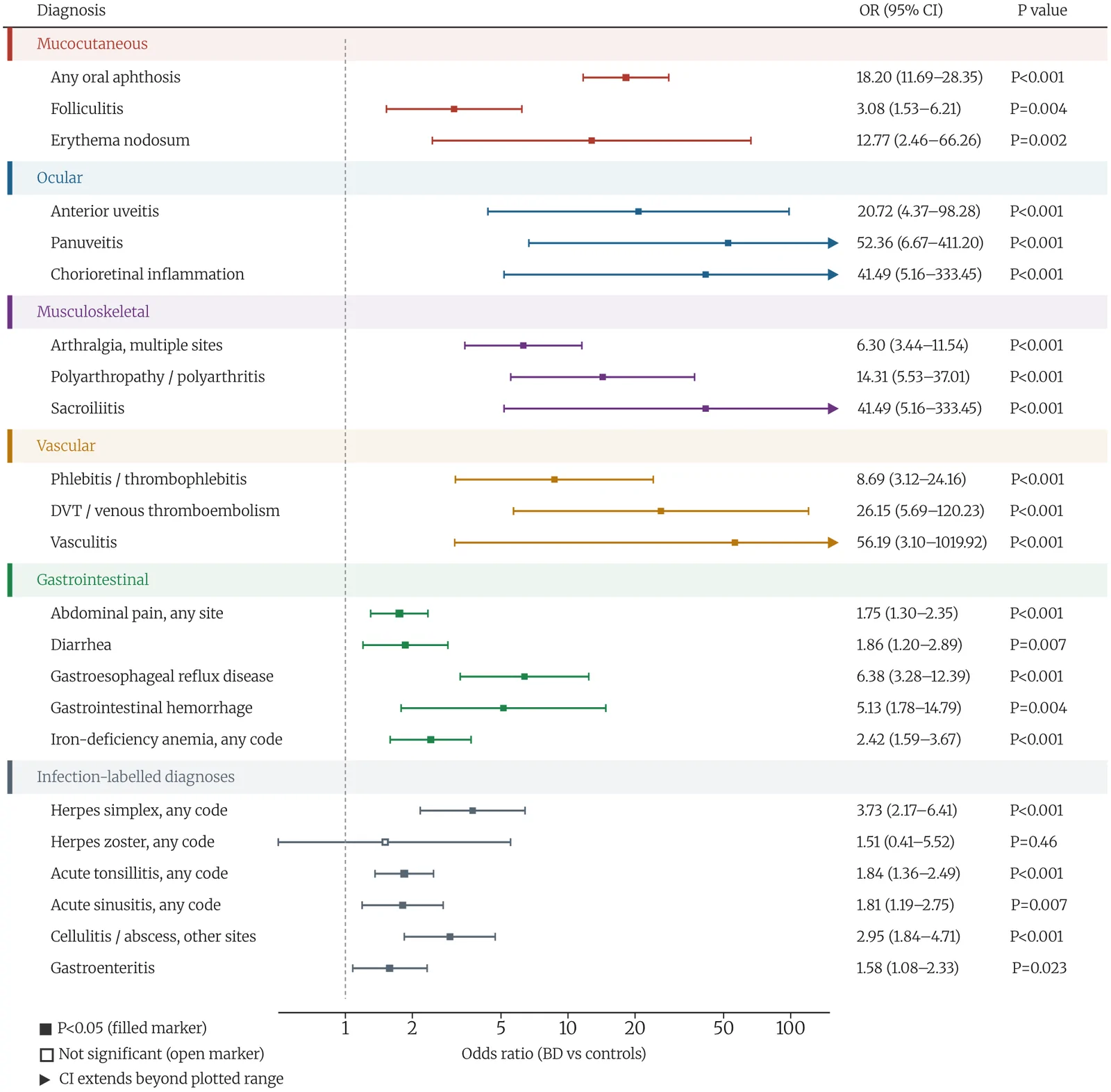
Clinical manifestations during the five-year prediagnostic period preceding Behçet’s disease diagnosis. Forest plot comparing the frequency of disease-related clinical manifestations recorded during the five years preceding the index date among patients subsequently diagnosed with Behçet’s disease and matched controls. Odds ratios (ORs) with 95% confidence intervals (CIs) are shown for manifestations grouped by organ system, including mucocutaneous, ocular, musculoskeletal, vascular, gastrointestinal, and infection-related diagnoses. Markers positioned to the right of the null value indicate manifestations that were more frequent among future patients with Behçet’s disease. Error bars represent 95% CIs, and arrows indicate confidence intervals extending beyond the plotted range. BD, Behçet’s disease; CI, confidence interval; DVT, deep venous thrombosis; GI, gastrointestinal; OR, odds ratio.

Musculoskeletal manifestations were likewise increased, including arthralgia involving multiple sites (11.0% vs 1.9%), inflammatory polyarthritis (7.3% vs 0.6%), and sacroiliitis (3.7% vs 0.1%) (all P<0.001). Similarly, vascular manifestations, including phlebitis or thrombophlebitis (4.6% vs 0.6%), venous thromboembolism (4.6% vs 0.2%), and vasculitis (2.3% vs 0%), were more frequent among future BD patients (all P<0.001).

Gastrointestinal manifestations were also more common during the prediagnostic period, including abdominal pain (45.9% vs 32.7%), diarrhea (14.2% vs 8.2%), gastroesophageal reflux disease (9.2% vs 1.6%), gastrointestinal hemorrhage (3.2% vs 0.6%) and iron-deficiency anemia (17.0% vs 7.8%) (all P ≤ 0.007).

Infection-labelled diagnoses occurred more frequently, particularly herpes simplex virus infection (11.0% vs 3.2%), acute tonsillitis (39.0% vs 25.8%), acute sinusitis (15.6% vs 9.3%), cellulitis or abscess (13.8% vs 5.1%) and gastroenteritis (18.8% vs 12.8%), whereas herpes zoster was comparable between groups (1.4% vs 0.9%; P = 0.46).

### Cumulative documented manifestations

When the entire available LHS record was considered, including the period after the index date, the documented frequencies of BD-related manifestations were substantially higher compared to the five-year prediagnostic window (**Table 3**). Mean follow-up after the index date was comparable between patients with BD and controls (11.9 ± 6.0 vs 11.8 ± 6.0 years, P = 0.67). Oral aphthosis was recorded in 61.0% of patients with BD compared with 11.8% of controls (OR 11.66, 95% CI 8.39-16.19), and erythema nodosum (6.0% vs 0.4%), folliculitis (21.1% vs 8.0%) and genital ulceration (2.3% vs 0.1%) were likewise more frequent (all P<0.001). The cumulative frequencies of ocular manifestations, including anterior uveitis (8.3%), posterior uveitis (5.5%) and panuveitis (8.7%), and of vascular manifestations, including venous thromboembolism (9.2%) and phlebitis or thrombophlebitis (9.6%), were approximately twice those recorded within the prediagnostic window (all P<0.001 vs controls) (**Table 3**).

**Table 3:** Cumulative documented clinical manifestations over the entire available follow-up.

Diagnosis	ICD-9 grouping/code	Behçet 5-year window*	Behçet entire record (n=218)	Controls entire record (n=1090)	OR (95% CI)	P value
Mucocutaneous						
Any oral aphthosis	528.2	79 (36.2%)	133 (61.0%)	129 (11.8%)	11.66 (8.39-16.19)	<0.001
Genital ulceration	616.5	3 (1.4%)	5 (2.3%)	1 (0.1%)	25.56 (2.97-219.92)	<0.001
Folliculitis	704.80	13 (6.0%)	46 (21.1%)	87 (8.0%)	3.08 (2.08-4.56)	<0.001
Erythema nodosum	695.2	5 (2.3%)	13 (6.0%)	4 (0.4%)	17.22 (5.56-53.33)	<0.001
Ocular						
Anterior uveitis	364.000	8 (3.7%)	18 (8.3%)	5 (0.5%)	19.53 (7.17-53.21)	<0.001
Posterior uveitis	363.202	5 (2.3%)	12 (5.5%)	1 (0.1%)	63.44 (8.20-490.54)	<0.001
Panuveitis	360.12	10 (4.6%)	19 (8.7%)	3 (0.3%)	34.59 (10.14-118.00)	<0.001
Chorioretinal inflammation	363	8 (3.7%)	15 (6.9%)	4 (0.4%)	20.06 (6.59-61.06)	<0.001
Musculoskeletal						
Arthralgia involving multiple sites	719.49	24 (11.0%)	63 (28.9%)	97 (8.9%)	4.16 (2.90-5.96)	<0.001
Unspecified polyarthropathy/polyarthritis	716.5	16 (7.3%)	35 (16.1%)	42 (3.9%)	4.77 (2.97-7.68)	<0.001
Sacroiliitis	720.2	8 (3.7%)	19 (8.7%)	28 (2.6%)	3.62 (1.98-6.61)	<0.001
Vascular						
Phlebitis and thrombophlebitis	451	10 (4.6%)	21 (9.6%)	19 (1.7%)	6.01 (3.17-11.38)	<0.001
DVT/other venous thromboembolism	453	10 (4.6%)	20 (9.2%)	16 (1.5%)	6.78 (3.45-13.31)	<0.001
Vasculitis	446	5 (2.3%)	11 (5.0%)	1 (0.1%)	57.87 (7.43-450.67)	<0.001
Gastrointestinal						
Abdominal pain, any site	789.0	100 (45.9%)	189 (86.7%)	779 (71.5%)	2.60 (1.72-3.93)	<0.001
Diarrhea	787.91	31 (14.2%)	89 (40.8%)	307 (28.2%)	1.76 (1.30-2.38)	<0.001
Gastroesophageal reflux disease	530.810	20 (9.2%)	37 (17.0%)	99 (9.1%)	2.05 (1.36-3.08)	<0.001
Gastrointestinal hemorrhage	578	7 (3.2%)	31 (14.2%)	69 (6.3%)	2.45 (1.56-3.85)	<0.001
Iron-deficiency anemia, any code	280	37 (17.0%)	86 (39.4%)	285 (26.1%)	1.84 (1.36-2.49)	<0.001
Infection-labelled diagnoses						
Herpes simplex, any code	054	24 (11.0%)	57 (26.1%)	122 (11.2%)	2.81 (1.97-4.01)	<0.001
Herpes zoster, any code	053	3 (1.4%)	21 (9.6%)	73 (6.7%)	1.49 (0.89-2.47)	0.149
Acute tonsillitis, any code	463	85 (39.0%)	165 (75.7%)	667 (61.2%)	1.97 (1.42-2.75)	<0.001
Acute sinusitis, any code	461	34 (15.6%)	106 (48.6%)	312 (28.6%)	2.36 (1.75-3.17)	<0.001
Cellulitis and abscess, other sites	682	30 (13.8%)	84 (38.5%)	274 (25.1%)	1.87 (1.38-2.53)	<0.001
Gastroenteritis	558.94	41 (18.8%)	118 (54.1%)	428 (39.3%)	1.83 (1.36-2.45)	<0.001

*Number of patients with Behçet’s disease with the diagnosis recorded during the five-year prediagnostic window, as presented in **Table 2**, shown for reference. Frequencies in the entire-record columns represent individuals with at least one corresponding diagnosis recorded at any time in the available LHS record, before or after the index date. P values refer to the comparison of the entire-record frequencies between patients with Behçet’s disease and controls. ICD-9, International Classification of Diseases, 9th revision. OR, odds ratio. CI, confidence interval. DVT, deep venous thrombosis.

### Autoimmune and infectious investigations during the prediagnostic interval

Autoimmune, infectious and genetic investigations were performed substantially more frequently among future patients with BD than among matched controls (**Table 4**). Despite this increased diagnostic activity, positivity rates for conventional autoimmune and infectious serologies were generally similar between groups. HLA-B51 testing was performed substantially more frequently among future patients with BD than controls (12.4% vs 0.1%; P<0.001). Among those tested, the HLA-B51 allele was detected in 33.3% of future patients with BD, whereas none of the tested controls carried the allele.

**Table 4:** Autoimmune and infectious investigations.

Investigation	Behçet tested n (%)	Controls tested n (%)	OR (95% CI)	P value
Panel A. Autoimmune investigations				
ANA	118 (54.1%)	91 (8.3%)	12.9 (9.2–18.1)	<0.001
RF	76 (34.9%)	56 (5.1%)	9.9 (6.8–14.5)	<0.001
Anti-CCP	15 (6.9%)	3 (0.3%)	26.8 (7.6–94.3)	<0.001
ANCA	72 (33.0%)	15 (1.4%)	35.3 (19.8–63.0)	<0.001
HLA-B51	27 (12.4%)	1 (0.1%)	153.9 (20.8–1139.6)	<0.001
Autoimmune serology positivity among tested patients				
ANA positivity	16/118 (13.6%)	14/91 (15.4%)	0.86 (0.40–1.87)	0.72
RF positivity	0/76 (0%)	0/56 (0%)	N/A	N/A
Anti-CCP positivity	0/15 (0%)	0/3 (0%)	N/A	N/A
ANCA positivity	0/72 (0%)	0/15 (0%)	N/A	N/A
HLA-B51 allele	9/27 (33.3%)	0/1 (0%)	N/A	N/A
Panel B. Infectious investigations				
HSV-1/2 IgM	13 (6.0%)	12 (1.1%)	5.7 (2.5–12.8)	<0.001
HSV-1/2 IgG	16 (7.3%)	13 (1.2%)	6.6 (3.1–14.2)	<0.001
CMV IgM	65 (29.8%)	153 (14.0%)	2.6 (1.9–3.6)	<0.001
CMV IgG	44 (20.2%)	100 (9.2%)	2.5 (1.7–3.7)	<0.001
EBV EBNA IgG	50 (22.9%)	101 (9.3%)	2.9 (2.0–4.2)	<0.001
Hepatitis C antibodies	68 (31.2%)	149 (13.7%)	2.9 (2.1–4.0)	<0.001
Infectious serology positivity among tested patients				
HSV-1/2 IgM positivity	0/13 (0%)	0/12 (0%)	N/A	N/A
HSV-1/2 IgG positivity	12/16 (75.0%)	10/13 (76.9%)	0.90 (0.16–5.01)	0.91
CMV IgM positivity	0/65 (0%)	0/153 (0%)	N/A	N/A
CMV IgG positivity	44/44 (100%)	100/100 (100%)	N/A	N/A
EBV EBNA IgG positivity	42/50 (84.0%)	89/101 (88.1%)	0.71 (0.27–1.86)	0.49
Hepatitis C antibody positivity	0/68 (0%)	0/149 (0%)	N/A	N/A

OR, odds ratio; CI, confidence interval; ANA, antinuclear antibody; RF, rheumatoid factor; CCP, cyclic citrullinated peptide; ANCA, antineutrophil cytoplasmic antibody; HLA, human leukocyte antigen; N/A, not applicable; HSV, herpes simplex virus; IgM, immunoglobulin M; IgG, immunoglobulin G; CMV, cytomegalovirus; EBV, Epstein-Barr virus; EBNA, Epstein-Barr nuclear antigen.

### Laboratory profile across the prediagnostic window

To characterize the evolution of laboratory abnormalities before diagnosis, the earliest and latest available measurements within the five-year prediagnostic interval were compared between future BD patients and matched controls (**Table 5**; **Figure 3**). Absolute monocyte count and the neutrophil-to-lymphocyte ratio demonstrated a temporal reversal. Both were lower among future BD patients at the early assessment but became significantly higher at the late assessment (monocytes: 0.40 vs 0.44 ×10^9^/L, P = 0.009, and 0.64 vs 0.58 ×10^9^/L, P = 0.025, respectively; NLR: 1.45 vs 1.49, P = 0.023, and 2.44 vs 2.09, P = 0.003, respectively). This transition was accompanied by increasing systemic inflammation. CRP was higher at both assessments and increased substantially during the late prediagnostic period (2.70 vs 2.10 mg/L, P = 0.020, and 10.70 vs 4.40 mg/L, P<0.001), whereas ESR, platelet count, total protein and globulin became significantly elevated only at the late assessment (all P<0.001).

**Table 5:** Trajectory of prediagnostic laboratory values (first vs. last measurement within the 5-year prediagnostic window).

Marker	Timepoint	Behcet n	Behcet median [IQR]	Control n	Control median [IQR]	P value
Inflammatory markers						
CRP (mg/L)	Early	161	2.70 [1.20-6.20]	391	2.10 [1.00-4.50]	0.02
	Late	161	10.70 [4.00-26.20]	391	4.40 [1.80-9.55]	<0.001
ESR (mm/h)	Early	148	14.50 [8.00-29.00]	281	13.00 [8.00-22.00]	0.151
	Late	148	29.00 [16.00-49.00]	281	17.00 [9.00-29.00]	<0.001
Platelet count (x10^9^/L)	Early	192	223.50 [188.00-263.00]	781	224.00 [189.00-265.00]	0.862
	Late	192	284.50 [243.00-338.00]	781	264.00 [222.00-311.00]	<0.001
NLR	Early	190	1.45 [0.97-1.81]	773	1.49 [1.11-1.91]	0.023
	Late	190	2.44 [1.69-3.30]	773	2.09 [1.55-2.96]	0.003
Non-albumin protein (g/dL)	Early	159	2.94 [2.62-3.29]	511	2.84 [2.62-3.08]	0.008
	Late	159	3.20 [2.89-3.44]	511	2.98 [2.75-3.25]	<0.001
Globulin-to-albumin ratio	Early	159	0.72 [0.61-0.79]	511	0.66 [0.60-0.74]	0.001
	Late	159	0.73 [0.65-0.81]	511	0.68 [0.61-0.76]	<0.001
CBC and white cell differential						
White blood cell count (x10^9^/L)	Early	192	5.72 [4.38-6.74]	781	6.04 [5.04-7.22]	0.003
	Late	192	7.98 [6.42-10.17]	781	7.78 [6.45-9.29]	0.087
Absolute neutrophil count (x10^9^/L)	Early	190	2.84 [2.13-3.83]	773	3.22 [2.52-3.93]	0.001
	Late	190	5.01 [3.49-6.25]	773	4.49 [3.49-5.87]	0.068
Absolute lymphocyte count (x10^9^/L)	Early	190	1.68 [1.39-2.16]	773	1.89 [1.50-2.34]	<0.001
	Late	190	2.49 [2.04-3.00]	773	2.43 [1.99-2.98]	0.508
Absolute monocyte count (x10^9^/L)	Early	190	0.40 [0.31-0.52]	773	0.44 [0.34-0.54]	0.009
	Late	190	0.64 [0.49-0.77]	773	0.58 [0.48-0.72]	0.025
Hemoglobin (g/dL)	Early	192	12.70 [11.50-13.93]	781	13.00 [11.70-14.40]	0.061
	Late	192	14.00 [12.90-15.20]	781	13.90 [12.90-15.10]	0.526
Iron-restricted and microcytic hematologic profile						
Serum iron (ug/dL)	Early	161	49.60 [32.80-72.80]	590	65.45 [44.02-88.38]	<0.001
	Late	161	98.50 [66.90-124.10]	590	95.50 [71.40-124.17]	0.942
Ferritin (ng/mL)	Early	113	22.00 [8.00-70.00]	403	27.00 [11.85-64.45]	0.195
	Late	113	48.10 [20.90-91.40]	403	38.35 [19.80-85.20]	0.341
Transferrin (mg/dL)	Early	126	259.50 [232.25-290.75]	359	264.00 [242.00-293.50]	0.088
	Late	126	286.50 [247.25-323.75]	359	285.00 [258.00-317.00]	0.659
MCV (fL)	Early	192	82.50 [77.60-85.45]	781	84.60 [80.80-87.40]	<0.001
	Late	192	85.50 [82.28-88.60]	781	87.20 [83.60-89.80]	0.005
Folic acid (ng/mL)	Early	88	4.60 [3.48-6.48]	315	5.90 [3.69-8.07]	0.015
	Late	88	6.78 [4.48-11.36]	315	7.07 [5.05-10.57]	0.767
Protein and immunoglobulin profile						
Albumin (g/dL)	Early	161	4.20 [3.90-4.30]	545	4.20 [4.00-4.40]	0.003
	Late	161	4.40 [4.30-4.60]	545	4.40 [4.20-4.60]	0.478
Total protein (g/dL)	Early	167	7.18 [6.80-7.56]	535	7.12 [6.85-7.44]	0.239
	Late	167	7.54 [7.22-7.91]	535	7.35 [7.12-7.67]	<0.001
Globulin (g/dL)	Early	153	2.90 [2.59-3.25]	463	2.83 [2.62-3.08]	0.055
	Late	153	3.27 [2.93-3.53]	463	3.00 [2.77-3.31]	<0.001
Immunoglobulin A (mg/dL)	Early	37	247.00 [183.00-308.00]	38	189.00 [150.25-221.00]	0.009
	Late	37	256.00 [183.00-319.00]	38	189.00 [152.50-228.25]	0.012
Immunoglobulin G (mg/dL)	Early	28	1163.50 [1050.75-1407.75]	18	1183.00 [1004.00-1464.75]	0.955
	Late	28	1188.00 [1064.00-1407.75]	18	1373.00 [1031.25-1488.50]	0.444
Immunoglobulin M (mg/dL)	Early	28	124.00 [73.75-155.50]	18	119.00 [69.50-180.00]	0.982
	Late	28	127.50 [94.00-166.25]	18	126.50 [94.25-180.00]	0.982

IQR, interquartile range; CRP, C-reactive protein; ESR, erythrocyte sedimentation rate; NLR, neutrophil-to-lymphocyte ratio; MCV, mean corpuscular volume. Additional erythrocyte and platelet indices are presented in **Supplementary Table S1**.

**Figure 3:**
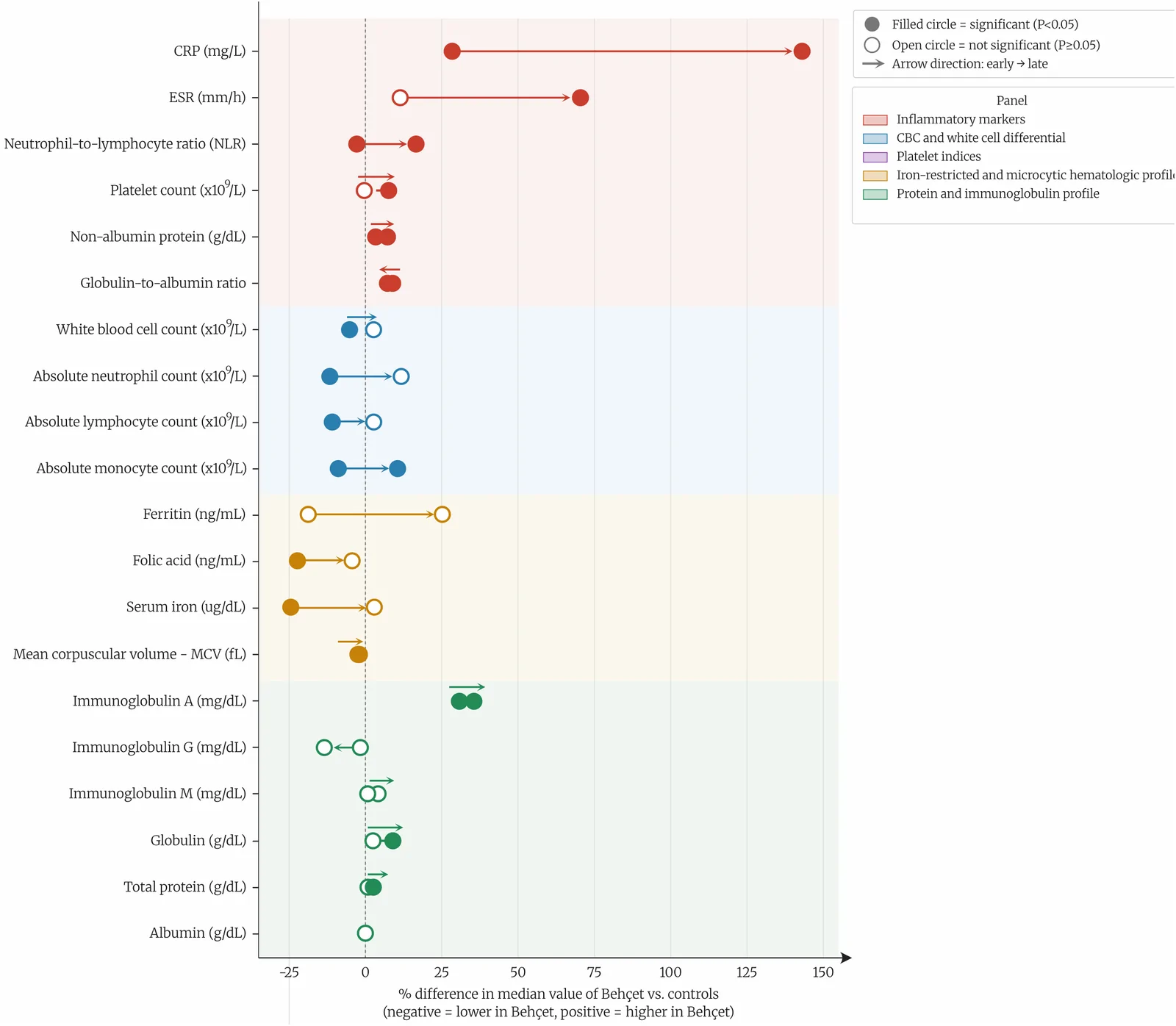
Longitudinal trajectory of prediagnostic laboratory markers in Behçet’s disease (BD). Each point represents the percentage difference between the median value in patients with BD (n=218) and the median value in matched controls (n=1,090) - calculated as (BD median − control median)/control median × 100 at two timepoints within the 5-year interval preceding diagnosis: the earliest (“Early”) and most recent (“Late”) value recorded for each marker. The dashed vertical line at 0% indicates no difference between groups; positive values indicate higher medians in BD, negative values indicate lower medians in BD. Arrows show the direction of change from the early to the late timepoint; filled circles denote P<0.05 while open circles denote P≥0.05. Acute-phase reactants (CRP, ESR, platelet count and globulin) showed little or no relative difference early but diverged sharply toward higher values in BD by the late timepoint, consistent with an emerging systemic inflammatory response as diagnosis approached. Leukocyte counts and iron-related parameters (white blood cell, neutrophil, lymphocyte and monocyte counts, serum iron and folic acid) instead showed a relative deficit early in BD that resolved by diagnosis. Notably, the neutrophil-to-lymphocyte ratio and absolute monocyte count reversed direction entirely, moving from significantly below control values early to significantly above them late. Immunoglobulin A was the only immunoglobulin showing a sustained, significant elevation among BD patients throughout the interval. BD, Behçet’s disease; CRP, C-reactive protein; ESR, erythrocyte sedimentation rate.

An iron-restricted and microcytic profile was also observed. Serum iron and folate were lower only at the early assessment, whereas MCV remained modestly lower at both the early and late assessments (82.5 vs 84.6 fL, P<0.001, and 85.5 vs 87.2 fL, P = 0.005, respectively). Among immunoglobulins, IgA was consistently higher among future patients with BD at both the early and late assessments (247 vs 189 mg/dL, P = 0.009, and 256 vs 189 mg/dL, P = 0.012, respectively), while IgG and IgM did not differ between groups. Additional erythrocyte and platelet indices are presented in **Supplementary Table S1**.

### Prediagnostic treatment exposure

During the five-year prediagnostic interval, future BD patients more frequently received colchicine (32.6% vs 0.9%, OR 52.2), systemic glucocorticoids (39.0% vs 13.0%, OR 4.27), including oral prednisone (32.1% vs 5.9%, OR 7.58) and immunosuppressive agents (10.6% vs 1.0%, OR 11.6) than matched controls (all P<0.001, **Supplementary Table S2**). Local oral corticosteroids (31.7% vs 4.5%) and ophthalmic corticosteroids (14.7% vs 3.9%) were also more frequently used. Among exposed future BD patients, treatment was initiated close to diagnosis, at a median of 0.1 years before the index date for colchicine and 0.5 years for oral prednisone and immunosuppressive agents.

## Discussion

Our findings suggest that BD is preceded by a clinically and biologically active prediagnostic phase. During the five years before diagnosis, future patients with BD accumulated mucocutaneous, ocular, musculoskeletal, vascular, gastrointestinal and infection-labelled manifestations. These clinical changes were accompanied by evolving laboratory abnormalities, including increase in monocyte counts, NLR, CRP, ESR, platelet count and serum proteins, together with persistent microcytic indices and selectively elevated IgA. Collectively, these findings may indicate that the prediagnostic interval represents an evolving multisystem disease state rather than a clinically silent period.

The spectrum and temporal distribution of clinical manifestations observed during the prediagnostic period are consistent with previous chronology studies of BD. Cohorts from Turkey, Iran, and Japan have consistently identified recurrent oral aphthosis as the earliest and most frequent manifestation, whereas ocular and vascular involvement generally develop several years later in the disease course (22–26, 33). Our findings are in keeping with this pattern. Oral aphthosis was the most prominent mucocutaneous manifestation, while ocular and vascular manifestations exhibited even stronger associations, consistent with their development closer to clinical diagnosis. By incorporating a matched population comparator, our study extends previous chronology studies by demonstrating that these early manifestations occur substantially more frequently than expected in the general population before the diagnosis of BD. Thus, the prediagnostic manifestations of BD are not simply the earliest symptoms recognized retrospectively, but may constitute a unique clinical signature that precedes fulfillment of conventional classification criteria.

The increased frequency of gastrointestinal diagnoses should not be interpreted as evidence of a high prevalence of intestinal BD. The recorded variables, including abdominal pain, diarrhea, gastroesophageal reflux disease, gastrointestinal bleeding and iron-deficiency anemia, represent nonspecific routine-care diagnoses documented before BD recognition. Their enrichment may instead reflect the broader symptomatic and diagnostic burden preceding BD diagnosis, including investigation of evolving systemic disease, bleeding, or causes unrelated to BD. The mechanisms underlying these associations cannot be established from the present data.

Previous studies of established BD have reported increased frequencies of metabolic comorbidities, including hypertension and diabetes mellitus (34). In the present study, however, neither condition was significantly more frequent during the prediagnostic period. This difference may reflect the relatively young age of our cohort and the fact that our analysis specifically evaluated the period preceding BD diagnosis rather than cumulative comorbidity during established disease.

Beyond the progressive accumulation of clinical manifestations, an important finding of our study is that the prediagnostic laboratory profile of BD evolved through two distinct biological phases rather than along a linear trajectory. Early in the prediagnostic interval, BD patients exhibited subtle hematologic and biochemical abnormalities, including lower leukocyte, neutrophil, and lymphocyte counts, reduced platelet size indices, hypoalbuminemia and persistently lower erythrocyte indices. By the time of diagnosis, many of these abnormalities resolved and were replaced by a conventional systemic inflammatory profile characterized by elevated CRP, ESR, platelet count, globulin, and red cell distribution width, together with reversal of monocyte count and the neutrophil-to-lymphocyte ratio. Although the biological basis of this transition remains uncertain, these findings may reflect evolution from an early phase of subclinical innate immune activation to acute-phase response that characterizes established inflammatory disease. To our knowledge, this temporal pattern has not previously been described in BD, likely because prior chronology studies lacked either a matched non-BD comparator, longitudinal laboratory assessment or both. If confirmed in independent cohorts, these findings suggest that the biological evolution of BD before diagnosis may be a dynamic process and include distinct prediagnostic phases rather than a simple linear escalation of inflammation.

The temporal reversal of monocyte count and the neutrophil-to-lymphocyte ratio deserves particular attention because it represents a qualitative shift rather than simply a change in the magnitude of inflammation. Both parameters were lower than in controls early in the prediagnostic interval but became significantly elevated immediately before diagnosis, suggesting a transition between biologically distinct phases. Although the mechanisms underlying this reversal remain uncertain, they are compatible with evolving activation of the innate immune system as clinical disease emerges. Similar increases in circulating monocytes and NLR have been consistently associated with disease activity in established BD, supporting the possibility that these markers reflect dynamic changes in immune activation during the transition from subclinical to clinically overt disease (35–37). Confirmation in prospective longitudinal cohorts, together with mechanistic studies is needed to determine whether this transition represents a reproducible feature of the early natural history of BD.

Unlike IgG and IgM, serum IgA remained consistently elevated throughout the prediagnostic interval. Although based on a relatively small subset of patients, this selective pattern is biologically plausible. Current models of BD pathogenesis suggest that dysregulated immune responses at mucosal barriers, driven by interactions between host genetic susceptibility and environmental or microbial exposures, contribute to disease initiation. Accordingly, recurrent oral aphthosis, genital ulceration, and gastrointestinal involvement are among the earliest clinical manifestations of BD (38–40). Consistent with this concept, IgA is the predominant immunoglobulin at mucosal barriers, where it reflects immune responses to luminal antigens and the resident microbiota (41, 42). The selective elevation of IgA observed in our cohort, together with the predominance of early mucosal manifestations, the increased frequency of HSV testing and diagnoses, and the near-universal absence of conventional systemic autoantibodies such as RF, anti-CCP antibodies, and ANCA, raises the hypothesis that the earliest immunologic changes in BD may preferentially involve dysregulated mucosal immune responses rather than classical systemic autoimmunity. Although speculative, this interpretation is consistent with accumulating evidence implicating barrier dysfunction and aberrant host-microbial interactions in BD pathogenesis (38–40). Given the limited number of patients with available immunoglobulin measurements, this observation should be considered hypothesis-generating and needs further confirmation in larger longitudinal cohorts incorporating detailed immunophenotyping.

Taken together, the convergence of disease-related manifestations, largely negative conventional serology and evolving laboratory abnormalities suggests that the prediagnostic phase of BD may be recognizable before formal diagnostic criteria are fulfilled. Rather than relying on a single diagnostic biomarker, earlier recognition may depend on identifying a characteristic prediagnostic footprint, a recurring multisystem pattern of clinical and laboratory features that emerges over time. This concept provides a rationale for developing and prospectively evaluating decision-support approaches that integrate clinical events and longitudinal laboratory trends into a composite risk signal. Such an approach may shorten the diagnostic delay by prompting earlier consideration of BD in patients with accumulating compatible features, although the present findings are hypothesis-generating and do not constitute a validated tool.

This study has several important strengths. By incorporating a matched general-population comparator, it addresses a key limitation of previous chronology studies. Furthermore, all BD cases underwent independent manual review by two board-certified rheumatologists with expertise in BD, ensuring a high degree of diagnostic accuracy beyond reliance on administrative coding alone. The integration of clinical diagnoses, healthcare utilization, diagnostic pathways, serological testing, and longitudinal laboratory data within the same cohort further enabled a comprehensive characterization of the prediagnostic phase.

Several limitations should also be acknowledged. Although all BD cases underwent rigorous manual adjudication, the retrospective design relies on data generated during routine clinical care rather than standardized research assessments. Furthermore, the analysis was restricted to the five years preceding the recorded BD diagnosis. Consequently, manifestations occurring earlier, particularly recurrent mucocutaneous manifestations that may precede diagnosis by many years, were not captured. The frequencies reported here therefore represent documented manifestations within the prespecified five-year prediagnostic interval and should not be interpreted as lifetime cumulative frequencies of BD manifestations. Similarly, the cumulative frequencies presented in **Table 3** include diagnoses recorded after the index date, which may partly reflect surveillance bias following BD diagnosis rather than the natural course of the disease alone. Even over the entire available record, the coded frequencies of several mucocutaneous features, particularly genital ulceration, were lower than those typically reported in clinical cohorts. This likely reflects documentation of these features in free-text clinical notes rather than as separately coded diagnoses. Some patients had already received anti-inflammatory or immunomodulatory therapy before the formal BD diagnosis. These treatments may have modified inflammatory and hematological measurements obtained during the prediagnostic interval, particularly those obtained close to diagnosis. For most parameters, including CRP, ESR and monocyte count, such effects would be expected to attenuate rather than exaggerate the observed differences, whereas glucocorticoid exposure may have contributed to the late increase in the neutrophil-to-lymphocyte ratio. The observed laboratory trajectories should therefore be interpreted as characterizing the clinical and biological state preceding formal diagnosis, which in a minority of patients already included treatment, rather than the untreated natural history of BD. In addition, some analyses were based on relatively small sample sizes, resulting in wide confidence intervals.

In conclusion, BD may be preceded by a clinically and biologically active prediagnostic phase characterized by a distinct footprint spanning multiple independent clinical and biological domains. These findings provide a rationale for developing and prospectively evaluating strategies aimed at recognizing this evolving disease state earlier, with the goal of shortening the diagnostic delay and facilitating earlier diagnosis.

## Data Availability

The datasets presented in this article are not readily available because The dataset contains individual-level electronic health record data and is subject to the data protection, privacy, and governance policies of Leumit Health Services (LHS). Therefore, the dataset cannot be made publicly available or shared directly by the authors. Requests to access the datasets should be directed to fadihh@gmail.com.

## Supplementary material

Supplementary Table 1 Additional erythrocyte and platelet indices during the five-year prediagnostic period (first vs. last measurement).

Supplementary Table 2 Medication exposure during the five-year prediagnostic period.
