# Supplementary material for: The prediagnostic period of Behçet’s disease: a nationwide population-based study
Source: Front Immunol. 2026 Oct 14;17:1970448. doi: 10.3389/fimmu.2026.1970448 (PMC13713727; doi:10.3389/fimmu.2026.1970448)
Supplement: Supplementary file 1 [file Table1.docx]

**Supplementary Table S1. Additional erythrocyte and platelet indices during the five-year prediagnostic period (first vs. last measurement)**

| Marker | Timepoint | Behcet n | Behcet median [IQR] | Control n | Control median [IQR] | P value |
| --- | --- | --- | --- | --- | --- | --- |
| Erythrocyte indices |  |  |  |  |  |  |
| RDW-CV (%) | Early | 192 | 12.90 [12.47-13.40] | 781 | 12.80 [12.40-13.40] | 0.974 |
|  | Late | 192 | 13.80 [13.20-14.90] | 781 | 13.50 [12.90-14.30] | <0.001 |
| MCH (pg) | Early | 192 | 27.40 [25.70-28.90] | 781 | 28.50 [26.80-29.50] | <0.001 |
|  | Late | 192 | 28.60 [27.50-30.20] | 781 | 29.30 [27.90-30.40] | 0.006 |
| Platelet indices |  |  |  |  |  |  |
| Mean platelet volume - MPV (fL) | Early | 191 | 10.50 [9.95-11.30] | 767 | 10.80 [10.20-11.50] | 0.002 |
|  | Late | 191 | 11.30 [10.60-12.15] | 767 | 11.40 [10.70-12.10] | 0.519 |
| Platelet distribution width - PDW | Early | 191 | 12.30 [11.25-14.10] | 767 | 12.90 [11.70-14.30] | 0.005 |
|  | Late | 191 | 14.10 [12.50-15.95] | 767 | 14.20 [12.80-15.75] | 0.625 |
| Platelet large cell ratio - P-LCR (%) | Early | 191 | 29.30 [24.75-36.20] | 767 | 32.10 [26.75-37.55] | 0.004 |
|  | Late | 191 | 35.90 [29.65-43.20] | 767 | 36.80 [31.30-42.35] | 0.525 |
| Plateletcrit - PCT (%) | Early | 89 | 0.26 [0.21-0.30] | 304 | 0.27 [0.21-0.30] | 0.526 |
|  | Late | 89 | 0.30 [0.28-0.40] | 304 | 0.30 [0.26-0.34] | 0.03 |

IQR - interquartile range. RDW-CV - red cell distribution width-coefficient of variation. MCH - mean corpuscular hemoglobin. MPV - mean platelet volume. PDW - platelet distribution width. P-LCR - platelet large cell ratio. PCT - plateletcrit.
