# Supplementary material for: The prediagnostic period of Behçet’s disease: a nationwide population-based study
Source: Front Immunol. 2026 Oct 14;17:1970448. doi: 10.3389/fimmu.2026.1970448 (PMC13713727; doi:10.3389/fimmu.2026.1970448)
Supplement: Supplementary file 2 [file Table2.docx]

**Supplementary Table S2. Medication exposure during the five-year prediagnostic period.**

| **Medication class** | **ATC code** | **Future BD (n=218)** | **Medication class (n=1090)** | **OR (95% CI)** | **Timing before index in BD*** |
| --- | --- | --- | --- | --- | --- |
| Colchicine-containing class | M04AC | 71 (32.6%) | 10 (0.9%) | 52.2 (26.3–103) | 0.1 [0.0–0.7] |
| Systemic glucocorticoids | H02AB | 85 (39.0%) | 142 (13.0%) | 4.27 (3.08–5.90) | 1.1 [0.2–2.8] |
| Oral prednisone | H02AB07 | 70 (32.1%) | 64 (5.9%) | 7.58 (5.18–11.1) | 0.5 [0.1–2.1] |
| Immunosuppressive agents | L04 | 23 (10.6%) | 11 (1.0%) | 11.6 (5.55–24.1) | 0.5 [0.0–2.5] |
| Local oral corticosteroids | A01AC | 69 (31.7%) | 49 (4.5%) | 9.84 (6.57–14.7) | 1.1 [0.3–2.9] |
| Ophthalmic corticosteroids | S01BA | 32 (14.7%) | 43 (3.9%) | 4.19 (2.58–6.79) | 1.2 [0.4–3.4] |

*Median [IQR] time in years between the first dispensing and the index date among exposed future BD patients. Systemic glucocorticoids include oral, intramuscular and intra-articular preparations. Immunosuppressive agents include methotrexate, azathioprine, leflunomide, cyclosporine and biologic agents. ATC - Anatomical Therapeutic Chemical classification. OR - odds ratio. CI - confidence interval. IQR - interquartile range.
